# Fetal Neuroprotective Strategies: Therapeutic Agents and Their Underlying Synaptic Pathways

**DOI:** 10.3389/fnsyn.2021.680899

**Published:** 2021-06-23

**Authors:** Nada A. Elsayed, Theresa M. Boyer, Irina Burd

**Affiliations:** ^1^Department of Gynecology and Obstetrics, Integrated Research Center for Fetal Medicine, Johns Hopkins University School of Medicine, Baltimore, MD, United States; ^2^Department of Neurology, Johns Hopkins University School of Medicine, Baltimore, MD, United States

**Keywords:** fetal neurodevelopment, fetal synaptopathy, magnesium sulfate, melatonin, N-acetyl-L-cysteine, DNAC

## Abstract

Synaptic signaling is integral for proper brain function. During fetal development, exposure to inflammation or mild hypoxic-ischemic insult may lead to synaptic changes and neurological damage that impairs future brain function. Preterm neonates are most susceptible to these deleterious outcomes. Evaluating clinically used and novel fetal neuroprotective measures is essential for expanding treatment options to mitigate the short and long-term consequences of fetal brain injury. Magnesium sulfate is a clinical fetal neuroprotective agent utilized in cases of imminent preterm birth. By blocking N-methyl-D-aspartate receptors, magnesium sulfate reduces glutamatergic signaling, which alters calcium influx, leading to a decrease in excitotoxicity. Emerging evidence suggests that melatonin and N-acetyl-L-cysteine (NAC) may also serve as novel putative fetal neuroprotective candidates. Melatonin has important anti-inflammatory and antioxidant properties and is a known mediator of synaptic plasticity and neuronal generation. While NAC acts as an antioxidant and a precursor to glutathione, it also modulates the glutamate system. Glutamate excitotoxicity and dysregulation can induce perinatal preterm brain injury through damage to maturing oligodendrocytes and neurons. The improved drug efficacy and delivery of the dendrimer-bound NAC conjugate provides an opportunity for enhanced pharmacological intervention. Here, we review recent literature on the synaptic pathways underlying these therapeutic strategies, discuss the current gaps in knowledge, and propose future directions for the field of fetal neuroprotective agents.

## Introduction

Perinatal brain development is an intricate process that begins early in fetal development and can be subject to numerous insults and delays. Perinatal brain injury is a leading maternal-fetal health issue that is often associated with preterm birth, cerebral palsy (CP), neurodevelopmental delay, and a spectrum of negative health sequelae (Burd et al., 2012; Duncan and Matthews, 2018; Hagberg et al., 2018). The mechanisms driving perinatal brain injury are varied, but are frequently tied to inflammation, infection, or hypoxia-ischemia, and subsequent cerebral white and gray matter cell death and damage (Novak et al., 2018; Stolp et al., 2019; McClendon et al., 2019; Mülling et al., 2020). Hypoxic-ischemic contribution to brain injury is more clearly defined clinically in term vs. preterm infants (Laptook, 2016; Gilles et al., 2018). Additionally, sex-specific differences in perinatal brain injury and response to treatment are often observed, with males at higher risk for perinatal brain injury compared to females (Bronson and Bale, 2014; Alexander et al., 2016; Thagard et al., 2017; Bolton et al., 2017; Motta-Teixeira et al., 2018; Al-Haddad et al., 2019a). Although the exact effect of these insults on synaptogenesis are unknown, increasing evidence links such insults to aberrant synaptic development and perinatal neurological deficits.

There is a concerning link between maternal infection and an increased risk of neuropsychiatric disorders in exposed *in utero* children (Lee et al., 2015; Ursini et al., 2018; Al-Haddad et al., 2019a,b; Kepinska et al., 2020). A Swedish birth cohort found that exposure to maternal infection, like influenza, increased the risk for autism and depression during the child’s lifetime (Al-Haddad et al., 2019a). Exposure to mild and ubiquitous infections, including a maternal urinary tract infection, can negatively impact fetal neurological development (Al-Haddad et al., 2019b). The gestational age of the fetus upon exposure to maternal infection may further dictate the severity of brain injury. There is a stronger correlation between maternal infection during the first or second trimesters compared to the third trimester or at delivery and the subsequent development of schizophrenia and other psychotic disorders in offspring (Khandaker et al., 2013). Some level of intrauterine inflammation is observed in about 20% of all pregnancies and in about 85% of very premature births (Elovitz et al., 2011). Other factors, such as genetics, may also contribute independently or synergistically to synaptic dysfunction. To date, several genome-wide association studies have identified links between neuropsychiatric disorders and synaptic structural and functional genes (Lee et al., 2012; Zoghbi and Bear, 2012; Schizophrenia Working Group of the Psychiatric Genomics Consortium et al., 2014; Schmidt-Kastner et al., 2020).

The prevention of perinatal brain injury has far reaching applications, such as decreasing societal neurological disease burden and improving the quality of life for affected mothers and neonates. This review summarizes the emerging evidence for the synaptic origins of fetal neurological disorders and details the use of several clinical and experimental neuroprotective agents.

### Fetal Synaptic Development and Excitotoxicity-Induced Synaptopathies

Within the dynamic context of fetal brain development, genetic, epigenetic, and environmental factors may contribute to the structure and function of fetal synapses as well as the development of synaptopathies (Fagiolini et al., 2009; Kundakovic and Ivana, 2017; Pozzi et al., 2018). In the human fetal brain, synaptogenesis normally begins around 5 weeks of gestation with development in an early cortical layer. Later in gestation, interaction with scaffolding cells such as astrocytes, microglia, radial glia, and oligodendrocytes leads to the initiation of neuronal migration. Functional synaptic transmission soon follows, and peak synaptogenesis is reached around 34 weeks of gestation with over 40,000 new synapses formed per second (Tau and Peterson, 2010). In addition, myelination appears to surge at 36 weeks of gestation (Hüppi et al., 1998). This critical period in fetal development ensures proper sensory, motor, and cognitive skills (Liberman et al., 2018). Neonates born or suffering injury before 34–36 weeks of gestation may thus be at higher risk for adverse neurodevelopmental outcomes compared to full-term neonates, due to deficits in synaptogenesis and myelination (Liberman et al., 2018; You et al., 2019). Postmortem tissue from preterm neonates confirms that the perinatal brain exhibits delayed synaptogenesis across several brain regions (Sarnat and Flores-Sarnat, 2016). Furthermore, there is a critical balance of apoptosis, or programmed cell death, in the developing brain that may be disrupted by preterm brain insult (Truttmann et al., 2020).

Glutamate acts as the main excitatory neurotransmitter in the central nervous system (CNS); however, glutamate is also implicated in fetal brain injury. Glutamate signals through both metabotropic and ionotropic post-synaptic receptors, initiating important cellular and molecular signaling cascades. Ionotropic glutamate receptors are membrane ion channel coupled and include N-methyl-D-aspartate (NMDA), α-amino-3-hydroxy-5-methyl-4-isoxazolepropionic acid (AMPA), and kainic acid (KA) receptors. Overexposure to glutamate and subsequent excess intracellular calcium influx, termed excitotoxicity, destroys neurons both *in vivo* and *in vitro* (Choi and Rothman, 1990; Lipton and Rosenberg, 1994; Mark et al., 2001; Park et al., 2010).

Following an initial mild hypoxic-ischemic injury or inflammatory trigger, the excitotoxic pathway culminates in neonatal brain injury (Silverstein et al., 1986, 1991; Hagberg et al., 1993; Burd et al., 2016). Impairment of oxidative metabolism results in ischemia, reducing oxygen delivery to cellular structures. These events, in turn, reduce neuronal depolarization. In a murine model of chorioamnionitis, this leads to a dependence on anaerobic metabolism and increases extracellular ATP release, likely as a direct response to inflammatory mediators (Lei et al., 2018).

**FIGURE 1:**
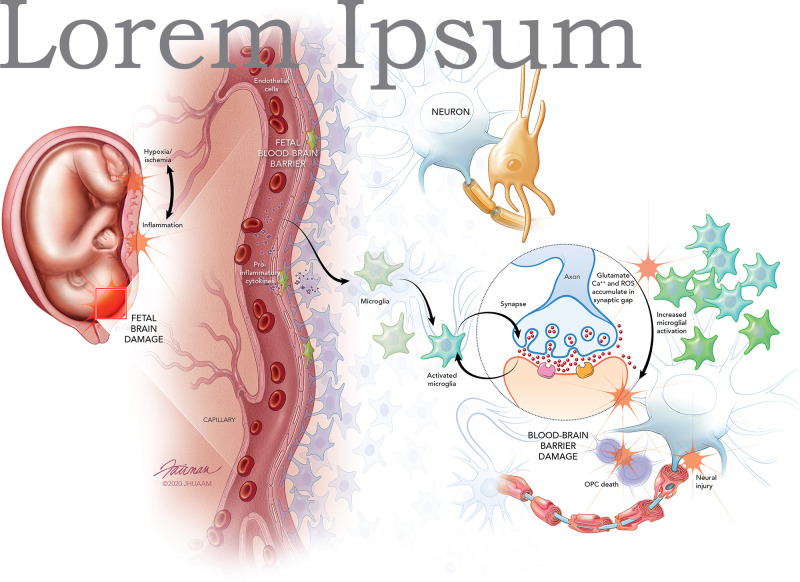
Fetal brain injury originates from inflammatory insult or mild hypoxic ischemic injury that triggers inflammation. Toll-like receptors on inflammatory cells of the placental membranes and decidua act as the inflammatory mediator leading to the induction and release of pro-inflammatory cytokines. The pro-inflammatory cytokines increase permeability of the developing fetal blood-brain-barrier (BBB), allowing recruitment of immune cells and increasing pro-inflammatory cytokines in the fetal brain. Fetal microglia are activated by the pro-inflammatory cytokines, and these activated microglia lead to the excessive release of glutamate. Overactivation of N-methyl-D-aspartate (NMDA), α-amino-3-hydroxy-5-methyl-4-isoxazolepropionic acid (AMPA), and kainate receptors expressed on neurons and oligodendrocyte precursor cells (OPCs) leads to the accumulation of glutamate in the synaptic gap. Calcium (Ca^2+^) and reactive oxygen species (ROS) also accumulate within the synaptic gap. Furthermore, NMDA receptors on microglia increase and exacerbate microglial activation. The overaccumulation of glutamate, Ca^2+^, and ROS leads to excitotoxic injury of neurons and OPCs culminating in fetal BBB damage, neural injury, and fetal brain damage.

Exposure to intrauterine inflammation may precipitate fetal neuroinflammation and the development of fetal-specific immune synaptopathies (Riazi et al., 2015; Pozzi et al., 2018; Figure 1). For instance, it is well established that intrauterine inflammation leads to activation of pro-inflammatory cytokines, which compromises the fetal blood brain barrier (BBB), leading to microglia activation and astrocyte signaling (Hagberg et al., 2015; Burd et al., 2016; Kelley et al., 2017). Microglial activation perpetuates pro-inflammatory cytokine expression and can lead to the build-up of reactive oxygen species (ROS), calcium, and glutamate-induced excitotoxicity (Vexler and Ferriero, 2001; Kaindl et al., 2012). Excessive intracellular calcium accumulation can also damage fetal oligodendrocyte precursor cells (Salter and Fern, 2005). Moreover, evidence indicates that maternal inflammation adversely impacts fetal neuronal morphology (Burd et al., 2010). The identification and mechanistic understanding of fetal-specific synaptopathies may provide the opportunity to develop fetal neuroprotective agents targeting and mitigating dysfunctional synaptic signaling.

Microglia, the resident immune cells of the CNS, are integral to maintaining CNS homeostasis, promoting neurogenesis, and mediating synaptic pruning (Paolicelli and Ferretti, 2017; Tay et al., 2018). In the early mouse postnatal brain, microglia make contact with synapses and engulf synaptic material (Paolicelli et al., 2011). Complement activation is key to this process of synaptic pruning, with complement molecules tagging synapses destined for removal by microglia (Tay et al., 2018; Druart and Le Magueresse, 2019). There is evidence that synaptic pruning begins prenatally, as microglia have been shown to accumulate in areas where synapses are nascent at the cortical plate-subplate junction (Verney et al., 2010). NMDA receptors are expressed on activated microglia and can perpetuate inflammation and cell death (Verney et al., 2010; Kaindl et al., 2012). Animal studies confirm that exposure to intrauterine inflammation leads to increased microglia activation and excitatory synaptic strength in the hippocampus (Kelley et al., 2017), with release of cytotoxic pro-inflammatory cytokines (Romero and Mazor, 1988; Yoon et al., 1997; Riazi et al., 2008; Welser-Alves and Milner, 2013). Aberrant microglia activation has been shown to continue into adulthood, particularly in the hippocampus (Kelley et al., 2017), which may result in long-term deficits in learning and memory if left untreated (Schaafsma et al., 2017; Bilbo and Stevens, 2017). This may be due to early priming of microglia in response to maternal inflammation, leading to loss of homeostatic function, increased sensitivity to future insults, and greater risk for neurodevelopmental and neuropsychiatric disorders (Tay et al., 2018). Inappropriate activation of the complement has also been implicated in neuropsychiatric disorders (Druart and Le Magueresse, 2019).

## Magnesium Sulfate

Magnesium sulfate (MgSO_4_) is a fetal neuroprotective agent that is administered in cases of imminent preterm birth. Originally used as a seizure prophylactic for women suffering from pre-eclampsia and later as a tocolytic agent to prevent preterm birth, MgSO_4_ has proven to have numerous beneficial effects for mother and fetus (Pritchard and Pritchard, 1975; Crowther et al., 2014; Ozen et al., 2019). Researchers first noted the neuroprotective properties of MgSO_4_ in the 1990s when infants of women who received MgSO_4_ during pregnancy had a lower incidence rate of CP (Nelson and Grether, 1995; Schendel et al., 1996; Paneth et al., 1997). Since then, multiple meta-analyses of randomized controlled trials of MgSO_4_ reveal that it is effective in decreasing the incidence of CP without increasing the risk of death in infants (Conde-Agudelo and Romero, 2009; Costantine and Weiner, 2009; Doyle et al., 2009; Shepherd et al., 2017).

The exact mechanism by which MgSO_4_ mediates its neuroprotective effects is not fully understood, although growing evidence indicates it is at least partially due to modulation of glutamatergic signaling. MgSO_4_ permeates freely across the BBB in neonatal swine (Rivera et al., 1991) and non-competitively blocks NMDA receptors, reducing calcium ion intake and preventing excitotoxic cell injury and death (Nowak et al., 1984; Marret et al., 1995). MgSO_4_ reduces both NMDA-induced and hypoxia-ischemia-induced rat brain injury and accelerates the differentiation of oligodendrocytes (McDonald et al., 1990; Thordstein et al., 1993; Kang et al., 2011; Itoh et al., 2016). The timing of MgSO_4_ administration is critical; across multiple animal models, MgSO_4_ leads to deleterious brain effects when administered after hypoxic insult as opposed to before the insult (Penrice et al., 1997; Galvin and Oorschot, 1998; Sameshima et al., 1999; Greenwood et al., 2000). This may be because administration of MgSO_4_ prior to a perinatal brain insult leads to preconditioning via mitochondrial resistance and reduced inflammation (Koning et al., 2018, 2019). In human neonates, administration of MgSO_4_ after birth for hypoxic-ischemic encephalopathy shows inconsistent long-term benefits and a trend toward increased neonatal mortality, underscoring the need to better understand the ideal timing of MgSO_4_ administration (Tagin et al., 2013). Dosing is another important consideration, as higher doses of MgSO_4_ may not confer greater neuroprotection and instead negatively affect fetal hemodynamics in animals and humans (Lecuyer et al., 2017).

MgSO_4_ is not without risks; in human neonates, MgSO_4_ can cause mild hypothermia, respiratory depression, feeding difficulties and longer neonatal intensive care unit (NICU) admissions (Lyell et al., 2007; Greenberg et al., 2013). Data from both preclinical and clinical studies show inconsistencies in long-term benefits of MgSO_4_ (Tagin et al., 2013; Galinsky et al., 2014). Despite these limitations, the evidence paints an overwhelmingly positive picture of this therapeutic agent and it continues to be used for fetal neuroprotection.

## Melatonin

Melatonin is an endogenous indolamine hormone synthesized and secreted by the pineal gland. It acts an antioxidant, neutralizing a number of ROS and reducing cellular damage (Tan et al., 1993, 2002; Reiter et al., 2009). Moreover, melatonin has anti-inflammatory properties, linked primarily to its reduction of nitric oxide levels and regulation of cytokine expression (Esposito and Cuzzocrea, 2010; Reiter et al., 2014).

In humans, the pineal gland does not fully develop until after birth, rendering the fetus dependent on its mother for melatonin. Maternal melatonin freely crosses the placenta to interact with ubiquitously expressed melatonin receptor MT1 in the fetus (Okatani et al., 1998; Thomas et al., 2002). Additionally, evidence indicates that the placenta itself contributes to melatonin synthesis (Lanoix et al., 2008). This provides a steady influence on the internal rhythm of the fetus along with protection from oxidative damage (Reiter et al., 2014; Motta-Teixeira et al., 2018). Preterm brain injury may interfere with this critical melatonin regulation, as data from a prospective multicenter study reveal that infants born before 34 weeks gestation have significantly lower levels of melatonin at birth and at 3 days of life compared to term infants (Biran et al., 2019).

Melatonin has low toxicity across a wide range of doses (Guardiola-Lemaitre, 1997; Jahnke et al., 1999) and has neuroprotective effects, regulating neuroexcitability and synaptic transmission in the hippocampus (Zeise and Semm, 1985; El-Sherif et al., 2002; Musshoff et al., 2002). Melatonin reduces impairments in long term potentiation and neuronal death in mouse models of Alzheimer’s disease and epilepsy (Liu et al., 2013; Ma et al., 2017), possibly by modulating AMPA subunit expression and preventing excitotoxic cell death (Soundarapandian et al., 2005; Ma et al., 2017).

Across multiple animal models of perinatal brain injury, melatonin has consistently been proven to be neuroprotective by increasing oligodendrocyte maturation and myelination repair, promoting axonal growth, and decreasing microglial activation after hypoxic, ischemic, inflammatory or drug-induced brain damage (Husson et al., 2002; Miller et al., 2005; Welin et al., 2007; Hutton et al., 2009; Olivier et al., 2009; Hamada et al., 2010; Kaur et al., 2010; Villapol et al., 2011). Melatonin is known to regulate neuronal generation and synaptic plasticity in the dentate gyrus, which is particularly susceptible to inflammatory and oxidative damage (Ramírez-Rodríguez et al., 2019). Furthermore, melatonin protects the dentate gyrus from neuroinflammation in neonatal rat models (Shah et al., 2017). Melatonin may offer this neuroprotection via activation of the SIRT1/Nrf2 signaling pathway, which is important for stimulating cell antioxidant responses (Shah et al., 2017; Lee et al., 2019a,b; Ren et al., 2019). Melatonin also reduces inflammatory damage and fetal death through reduction of hypoxic and oxidative stress (Chen et al., 2006; Wang et al., 2011; Lee et al., 2019a,b). Maternal melatonin deprivation during gestation and lactation in a rat model leads to neurobehavioral and neurodevelopmental deficits, primarily seen in male fetuses and rescued by administration of melatonin (Motta-Teixeira et al., 2018). An important consideration in future clinical use of melatonin is excipient influence, as observed neuroprotection in neonatal piglet asphyxia models differ based on excipient use (Robertson et al., 2020).

In human neonates, administration of melatonin for sepsis leads to decreased lipid peroxidation products and improved clinical outcome (Gitto et al., 2001). Multiple clinical trials of melatonin are currently underway to assess its ability to provide neuroprotection for growth restricted fetuses and in cases of neonatal asphyxia (Miller et al., 2014; Aly et al., 2015; Wilkinson et al., 2016). Preliminary results indicate that melatonin reduces placental oxidative stress in pregnancies complicated by fetal growth restriction (Miller et al., 2014) and improves neurodevelopmental outcomes in neonates with perinatal asphyxia (Aly et al., 2015). Melatonin shows a wealth of potential as a fetal neuroprotective agent, and the next decade will likely usher in more data supporting the use of this molecule in pregnancy.

## N-Acetyl-L-Cysteine

N-acetyl-L-cysteine (NAC) is an acetylated amino acid approved by the United States Food and Drug Administration for the treatment of acetaminophen toxicity (Scalley and Conner, 1978; Smilkstein et al., 1988). Recently, NAC was found to have psychiatric and neurological applications and is experimentally used for the treatment of addiction, schizophrenia, bipolar disorder, and obsessive-compulsive disorder (Lafleur et al., 2006; Berk et al., 2008, 2012; Gray et al., 2010). Neuroinflammation is shown to disrupt synaptogenesis (Riazi et al., 2015; Pozzi et al., 2018), and NAC may exert therapeutic benefit through the decrease of synaptic glutamate release mitigating subsequent excitotoxic neurological damage (Dean et al., 2011).

NAC’s therapeutic properties stem from its action on the cystine-glutamate antiporter system and as an antioxidant to regulate the neuroinflammatory response (Dean et al., 2011; Durieux et al., 2015). The cystine-glutamate antiporter, system x_c_^–^, is a highly conserved heterodimeric amino acid transporter that exchanges extracellular cystine for intracellular glutamate across the plasma membrane. In animal models, regional distribution studies demonstrate the presence of the antiporter protein in the meninges, cortex, hippocampus, striatum, and cerebellum of embryonic and adult rat brains (Shih et al., 2006). Developmental age impacts the expression of the transporter, and immature neurons exhibit greater system x_c_^–^ expression compared to mature neurons (Murphy et al., 1990; Shih et al., 2006). Within the CNS, system x_c_^–^ is found in neurons and glial cells, such as astrocytes (Shih et al., 2006). It provides an essential non-vesicular pathway for extracellular glutamate release (Bridges et al., 2012; Lewerenz et al., 2013) and may inhibit the clustering of post-synaptic glutamate receptors (Augustin et al., 2007). Cysteine, the oxidized form of cystine, also acts as the rate-limiting substrate in the production of the tripeptide endogenous antioxidant glutathione (GSH) (Gunduz-Bruce et al., 2012). Astrocytes play a critical role in cystine uptake to supply the surrounding neurons with cysteine for GSH production (Wang and Cynader, 2002). System x_c_^–^ mediates the link between the release of excitatory neurotransmitters, in the form of glutamate, and the modulation of oxidative stress through the generation of GSH.

NAC increases the availability of cysteine within the extracellular space. This increase leads to the production of GSH and regulates glutamate release through system x_c_^–^. Stimulation of the antiporter system increases glutamate within the extrasynaptic space and activates presynaptic inhibitory metabotropic glutamate receptors, mGluR2/3. Ultimately, this reduces the synaptic release of glutamate (Baker et al., 2002; Dean et al., 2011). Analysis of plasma GSH levels show oral NAC administration yields a greater GSH fraction compared to the administration of GSH or L-cysteine (Borges-Santos et al., 2012; Schmitt et al., 2015).

The clinical utility of NAC shows promise in clinical trials (Amin et al., 2008; Jenkins et al., 2016). In a double-blind Phase 1/2 trial of NAC as a neuroprotective agent in cases of maternal chorioamnionitis, IV NAC was maternally administered every 6 h until delivery. After delivery, IV NAC was administered to neonates every 12 h for five doses (Jenkins et al., 2016). During the study, no maternal or neonatal adverse events associated with NAC were observed. While neonatal cerebrovascular measures did not differ between the control and treatment groups, inflammatory cytokines quantified in maternal serum, cord serum, and neonatal cerebral spinal fluid samples improved following NAC administration (Jenkins et al., 2016). Both serum fibroblast growth factor 2 and IL-17 were reduced in the NAC treatment group compared to the control group. NAC also shows improved neuroprotective benefits compared to melatonin, another free radical scavenger (Wang et al., 2007). These findings demonstrate the translational ability of NAC for use as a fetal neuroprotective agent; however, larger clinical studies are needed to fully evaluate its safety and efficacy.

The impact of the physiological changes of pregnancy, including hemodynamic changes, on NAC pharmacokinetics remains largely unknown (Pavek et al., 2009). In healthy non-pregnant individuals, the half-life of oral NAC is 6.25 h, while the half-life of IV NAC is 5.58 h (Olsson et al., 1988; Prescott et al., 1989). However, a study of IV maternal NAC administration reports the half-life of NAC as 1.2 h at a dosage of 100 mg/kg within the pregnant population (Wiest et al., 2014). Additionally, differences in the bioavailability of oral NAC administration compared to maternal IV NAC administration should be further characterized.

The optimal timing of NAC administration to prevent fetal neurological damage remains an area of ongoing research. In murine models, NAC shows therapeutic benefit when administered prenatally or within 1 day of birth (Lanté et al., 2008). No neurological benefits of NAC are observed if treatment is delayed 2 days after delivery. Since NAC crosses the placental barrier and BBB in both humans and mice, it likely exerts its benefits through restoring the fetomaternal oxidative balance and decreasing the excitotoxic effects of excess glutamate (Horowitz et al., 1997; Lanté et al., 2008; Wiest et al., 2014). Additional studies are needed to provide guidance about the optimal timing of maternal NAC administration to achieve maximal therapeutic benefits for the fetus in humans.

### Dendrimer-Bound N-Acetyl-L-Cysteine

While NAC has been found to be therapeutically usefully, NAC has presented with many adverse effects at the recommended dosage. Conjugating NAC with a vehicle, such as a dendrimer, may improve the bioavailability and tolerability of the drug (Kannan et al., 2012). The dendrimer-bound NAC conjugate (DNAC) shows promise in optimizing drug administration and mitigating deleterious medication side-effects. This demonstrates the improved possibilities of targeted fetal neuroprotective therapies. DNAC is a polyamidoamino (PAMAM) dendrimer conjugated with NAC (Lei et al., 2017). Disulfide linkers, cleavable by exposure to GSH, enable controlled intracellular release of NAC (Kurtoglu et al., 2009). In a randomized trial of the addition of NAC to 17-hydroxyprogesterone treatment in women with bacterial vaginosis and at high-risk of preterm birth, 11.4% of participants discontinued NAC due to nausea, vomiting, and/or gastric disruptions (Shahin et al., 2009). Therefore, there is a clear need to develop more tolerable alternatives to improve translational feasibility and treatment compliance.

A dendrimer-based complex therapy also presents the opportunity to improve drug delivery and to optimize drug functionality *in vivo* (Madaan et al., 2014). Dendrimers enhance solubility, stability, and the bioavailability of drugs through a three-dimensional branched polymer scaffolding surrounding the drug molecule (Chauhan, 2018). Varying surface groups and the charge of terminal moieties can modulate drug toxicity and tolerability (Gillies and Frechet, 2005; Janaszewska et al., 2019). Nanostructured molecules improve the permeability of drugs across physiological barriers, such as epithelial and mucosal barriers, and they enable targeted drug delivery to the specific sites of disease and injury (Kannan et al., 2014).

The surface groups of dendrimers are modified to improve targeted drug delivery to areas of interest (Wang et al., 2009; Zhang et al., 2016). The modification of dendrimers is especially relevant when considering their ability to cross the placental barrier and undergo selective fetal absorption (Menjoge et al., 2011; Burd et al., 2014). In an intra-amniotic delivery system of the neutrally charged hydroxyl-terminated (D-OH) dendrimer, the dendrimer was transported across the placental barrier and absorbed by the rabbit fetus. This showed superiority to the negatively charged carboxyl-functionalized dendrimer (D-COOH) when crossing the BBB of the fetus. The dendrimer was systemically administered to the mother following an inflammatory insult, and the D-OH dendrimer selectively localized in activated fetal microglia (Zhang et al., 2016). This localization continued into the postnatal period providing a continued level of protection.

Postnatal administration of DNAC shows promise in attenuating neonatal neurological injury following maternal inflammation (Kannan et al., 2012). This appears in contrast with the lack of efficacy of postnatal free NAC administration (Lanté et al., 2008). In a rabbit model of CP, DNAC administrated intravenously on postnatal day one improved neurological function of kits and decreased markers of neuroinflammation. CP kits in the DNAC treatment group also showed increased levels of myelin basic protein compared to CP kits that received phosphate buffered saline. Markers of oxidative stress to lipids, proteins, and RNA also decreased within the DNAC treatment group (Kannan et al., 2012).

## Conclusion

There remains a paucity of data about fetal neuroprotective therapeutic agents and their underlying synaptic pathways. Given the growing prevalence of perinatal neurological injury, expanding the armamentarium of fetal neuroprotective agents is a pressing issue for researchers. Newer agents with potential to modulate neuronal plasticity and treat neonatal hypoxia-ischemia, such as erythropoietin, should be further explored (Oorschot et al., 2020). Future studies should also address sex-appropriate therapeutic agents to better characterize which neonates derive benefit from specific treatments.

## Author Contributions

TB and NE contributed equally to the writing of this manuscript. IB reviewed and edited the manuscript. All authors contributed to the article and approved the submitted version.

## Conflict of Interest

The authors declare that the research was conducted in the absence of any commercial or financial relationships that could be construed as a potential conflict of interest.
